# Reduced Pharmacological Intervention of Prehospital Services for Acute Alcohol Intoxication during the COVID-19 Pandemic in A Large District of Southern Italy

**DOI:** 10.3390/jcm13113057

**Published:** 2024-05-23

**Authors:** Arcangela Giustino, Annamaria Natola, Giovanni Savoia, Maria Antonietta De Salvia, Carmine Finelli

**Affiliations:** 1 Department of Precision and Regenerative Medicine and Ionian Area, Medical School, University of Bari “Aldo Moro”, Piazza Giulio Cesare, 11, 70124 Bari, Italy; arcangela.giustino@uniba.it (A.G.); mariaantonietta.desalvia@uniba.it (M.A.D.S.); 2 Operations Centre of Emergency Services (118)—Policlinico Hospital, Piazza Giulio Cesare, 11, 70124 Bari, Italygiannisavoia@gmail.com (G.S.); 3 Department of Internal Medicine, Hospital “A. Maresca”–ASL Naples 3 Sud, Via Montedoro, 1, 80059 Torre del Greco (Naples), Italy

**Keywords:** emergency service, acute alcohol intoxication, triage, pharmacological treatment, public health

## Abstract

**Background** Stress during a pandemic increases the risk of alcohol consumption, which may require pharmacological management. **Methods** An observational single-center retrospective study was conducted from 1 January 2018 to 31 December 2021, and divided into 2-year periods (2018–2019 and 2020–2021). This study focused on calls to one of the emergency departments (EDs) of seven hospitals in the Bari (Italy) metropolitan area for patients requiring emergency services (ESs) who were either admitted or not admitted, due to their refusal. **Results** A 30% reduction in emergency calls for alcohol-related issues and a 41.17% reduction in calls for patients who refused to be admitted to the ED were observed during the pandemic. During the pandemic, an inverse association was found between pharmacological treatment and number of calls coded green (non-critical) and yellow (fairly critical) in patients admitted to EDs. An inverse association was also found for calls coded green in patients not admitted to EDs and pharmacological treatment. Metadoxine was administered in almost all alcohol-related emergencies, primarily in conjunction with drugs acting on the gastrointestinal tract, irrespective of age, the period considered, and whether patients were admitted or not admitted to the ED. **Conclusions** ES is the first and only out-of-hospital service encountered by numerous patients with alcohol-use disorders who refuse to be admitted to the ED. These patients should be directed by ES personnel to a multidisciplinary program to receive treatment for drinking, improve their quality of life, and reduce sanitation costs.

## 1. Introduction

Europe has the highest alcohol consumption worldwide [1,2,3].

The 2020 Italian Ministry of Health Report highlighted that 14.3% of the young adult Italian population is at increased risk of developing alcohol-related disorders requiring treatment [4].

It is of significant concern that the increasing drinking style of alcohol consumption over the last decades, characterized by a “wet habit of socialization,” has prompted larger percentages of young and adult individuals to adopt risky alcohol consumption habits. This has resulted in more individuals making use of sanitary emergency services (ESs) in instances of alcohol intoxication [5,6,7,8].

At the beginning of 2020, the highly contagious severe acute respiratory syndrome coronavirus 2 (SARS-CoV-2), induced severe infective pathology worldwide, starting from China and extending to Europe from Northern Italy. This became known as the coronavirus of 2019 (COVID-19) pandemic.

The concomitant circumstances of health emergency requirements, due to both risky alcohol consumption and/or COVID-19 viral infection during the pandemic, may have influenced management of the therapeutic approach provided by the 118 telephone number ESs. These ESs attempted to guarantee the availability of sanitary services to assist individuals affected by the pandemic and/or any health-threatening emergency by providing first aid.

Moderate acute alcohol intoxication does not require pharmacological treatment; however, severe intoxication requires drugs [9].

Therefore, our study aimed to characterize the 118 ESs during the 2 years of the COVID-19 pandemic with respect to the previous 2 years, specifically with regard to alcohol intoxication.

In particular, we characterized the pharmacological treatments provided by the 118 ESs to individuals in alcohol emergency-threatening conditions, both in cases where admission to the emergency department (ED) was accepted or refused.

## 2. Materials and Methods

### 2.1. Study Design

An observational single-center retrospective study was conducted from 1 January 2018 to 31 December 2021, and divided into 2-year periods: 2018–2019 was considered as the pre-pandemic period and 2020–2021 was considered as the COVID-19 pandemic period. The aim was to evaluate the effect of the lockdown period (March–May 2020 in Italy, during the first wave of the COVID-19 pandemic) as well as the effect of the second wave of the COVID-19 pandemic (from October 2020, which induced the Italian government to introduce restrictions on movement and social life).

The study involved calls to the “118” emergency number for patients requiring emergency care in the Bari (Italy) large metropolitan area, who were then admitted to one of the EDs of seven hospitals in the area and those who, although requiring ESs, refused to be admitted to the ED. The group of patients requiring ESs and who were admitted to ED are henceforth referred to as the “ED group”, and patients who required ESs but refused to be admitted to the ED are henceforth referred to as the “NED group”. In the present study, the participants were ≥11 years and required emergency care for alcohol-related conditions, as noted in the electronic health records compiled by emergency doctors and nurses during ESs. The conditions included alcoholism, alcohol abuse, alcoholic coma, drunkenness, acute alcohol intoxication with or without drug abuse, and alcohol breath.

This study was approved by the Local Ethics Committee of the University Hospital (Italy). Retrospective data analysis was performed based on the Italian Data Protection Authority’s General Authorization to Process Personal Data for Scientific Research Purposes (authorization number 15260/COMET, 15 February 2023).

### 2.2. Collection of Data

Data were extracted from electronic health records compiled by emergency doctors and nurses during ESs. Among the information recorded for each emergency call, we analyzed the following: (a) demographic data (age and sex); (b) triage color code (red, yellow, green, white, and black) based on the Emergency Severity Index (ESI) [10]; (c) diagnosis of apparent pathologies; and (d) medicines administered and the route of administration.

The administered medicines were grouped as follows: those acting on the central nervous system (CNS; benzodiazepines and chlorpromazine, haloperidol, and clotiapine); those acting on the gastrointestinal (GI) system (pantoprazole, ranitidine, metoclopramide, acetylcysteine and levosulpiride); those acting on the respiratory system (RS; salbutamol, corticosteroids, theophylline, oxygen, and chlorpheniramine); those considered to be pharmacologically ideal antidotes (naloxone [Nal] and/or flumazenil [Flu]); and those acting on other systems (pain killers, antispasmodics, insulin, and glucagon).

### 2.3. Statistical Analyses

Statistical analyses were conducted using StatSoft 5.5 (Tulsa, Oklahoma, United States) and R version 4.0.3 software. Categorical data are reported as frequencies. The Pearson’s chi-square test was used to analyze independence between categorical variables. The odds ratios (ORs) and 95% confidence intervals (CIs) were calculated. OR > 1 with *p* < 0.05 was reported as a risk factor. Statistical significance was set at *p* < 0.05.

## 3. Results

The age distribution and demographic characteristics of patients requiring emergency care for alcohol-related conditions across the 2-year periods (2018–2019 and 2020–2021) are presented in Table 1.

The percentages of alcohol-related emergency calls with respect to emergency calls for all other reasons were significantly higher in 2018–2019 compared to 2020–2021: 1.63% (3710/226,996) and 1.18% (2601/219,466), respectively (Pearson’s chi-square test, chi squared = 161.59, df = 1, *p* < 0.05).

No sex or age data were recorded for 33 and 384 service users, respectively. Therefore, those data were not included in the analysis. Of the total calls, 4869 (77.15%) emergency calls involved male patients.

As shown in Table 1, a reduction of nearly 30% in emergency calls for alcohol-related issues during the pandemic period (2020–2021) was observed compared to the pre-pandemic period (2018–2019). Interestingly, the percentage reduction in emergency calls between the periods under study per age group showed the highest value for the 25–44 age group, followed by the 18–24 age group. Low reductions were observed in the most fragile groups, such as minors (11–17 years of age) and older adults (≥65 years of age), who may be more susceptible to alcohol toxicity.

However, we observed a low reduction also for subjects aged 45–64 years, likely because of alcohol-related comorbidities, which made them unable to avoid ESs even during the pandemic period (2020–2021). Notably, 35.04% (1300/3710) of emergency calls for alcohol-related issues did not end in the ED in 2018–2019.

During the pandemic period, an even higher percentage of patients, 41.17% (1071/2601), refused to be admitted to the ED (Pearson’s chi-squared test, chi-squared= 24.54, df = 1, *p* < 0.05).

Based on this finding, we investigated how and if the decision to admit patients pharmacologically treated by ESs to the ED would be affected by the pandemic.

Therefore, our study compared two different groups, namely patients to be admitted (ED group) or not admitted (NED group) to the ED, across two periods: the pre-pandemic (2018–2019) and pandemic (2020–2021) periods.

Evaluation of the percentages of emergency calls for patients pharmacologically treated by ESs showed lower values in the ED groups both during the pre-pandemic (25.08% vs. 32.05%, Pearson’s chi-squared test, chi-squared = 20.56, df = 1, *p* < 0.05) and pandemic (17.07% vs. 25.09%, Pearson’s chi-squared test, chi-squared = 25.00, df = 1, *p* < 0.05) periods, with respect to the NED groups.

Moreover, lower percentages of emergency calls for patients pharmacologically treated were observed when we compared the ED groups (25.08% vs. 17.07%, Pearson’s chi-squared test, chi-squared= 35.03, df = 1, *p* < 0.05) and the NED groups (32.05% vs. 25.09%, Pearson’s chi-squared test, chi-squared = 13.85, df = 1, *p* < 0.05) before and during the pandemic, suggesting that a reduced number of patients were pharmacologically treated during the pandemic, whether or not they were admitted to the ED (Table 2).

It would be interesting to investigate whether the reduced percentage of calls for pharmacologically treated patients was due to changes in the severity of the condition of patients requiring ESs. Therefore, we analyzed how calls for patients requiring ESs and pharmacological treatment were classified based on triage in ED and NED, period, and age groups. The calls were classified as green for noncritical cases, yellow for fairly critical cases, and red for very critical cases.

As for calls coded “green”, statistical analysis showed that there was an inverse association between pharmacological treatment and the periods under consideration in both patient groups to be admitted (ED group with green code: OR = 0.53, 95% CI (0.41–0.68), z = 4.85, *p* < 0.05) and not admitted to ED (NED group with green code: OR = 0.71, 95% CI (0.59–0.86), z= 3.56, *p* < 0.05) (Table 3).

As for calls coded “yellow”, statistical analysis showed that there was an inverse association between pharmacological treatment and periods under consideration only in calls of the patient group to be admitted to ED (ED group with yellow code: OR = 0.47, 95% CI (0.37–0.59), z= 6.2, *p* < 0.0001). However, no such association was found in the calls of the patient group not to be admitted to ED (NED group with yellow code: OR = 0.63, 95% CI (0.31–1.27), z = 1.3, n.s.).

No association was found in calls coded “red” for both patient groups (ED group with red code: OR = 0.94, 95% CI (0.53–1.68), z = 0.202, n.s.; NED group with red code: OR = 0.11, 95% CI (0.0012–10.27), z = 0.95, n.s.).

To evaluate whether the findings reported in Table 3 would include all age groups, Table 4 reports the percentages of calls for patients pharmacologically treated by age group, period, and code of triage in the two groups under investigation (ED and NED groups).

A reduction in the number of calls coded “green” for patients pharmacologically treated with ES was observed in all age groups in both the ED and NED groups when the two time periods under examination were compared (Table 4).

A reduced number of calls coded “yellow” for patients pharmacologically treated by ESs was observed in all age groups when comparing the two periods under examination in the ED. This reduction was not observed in the NED group (Table 4).

An increase in the percentage of calls coded “red” for patients pharmacologically treated by ESs was observed in the ED groups up to 24 years of age during the pandemic period compared to the pre-pandemic period (Table 4).

We then calculated the percentages of emergency calls for alcohol-related issues for patients pharmacologically treated and admitted (ED group) or not admitted to the ED (NED group) during the pre-pandemic and pandemic periods by age group (Table 5).

For the 11–17 years of age group, a significant reduction was observed only between the percentages of calls for patients not admitted to the ED (NED group) across the two time periods analyzed (2018–2019 vs. 2020–2021) (46.4% vs. 25.9%, Pearson’s chi-squared test, chi squared= 4.99, df = 1, *p* < 0.05).

For the 18–24 years of age group, a significant reduction was observed in the percentage of calls for patients not admitted to the ED (ED group) during 2018–2019 with respect to the COVID-19 period (2020–2021) (33.9% vs. 19.2%, Pearson’s chi-squared test, chi squared = 12.40, df = 1, *p* < 0.05).

Moreover, a significant increase in the percentage of calls for patients not admitted to the ED (NED group) with respect to the percentage of calls for patients admitted to the ED (ED group) between the 2-year periods analyzed was recorded (33.3% vs. 19.2%, Pearson’s chi-squared test, chi squared= 8.49, df = 1, *p* < 0.05).

For the 25–44 years of age group, a significant increase was observed between the percentages of calls for patients admitted to the ED (ED group) or not admitted (NED group) during both 2018–2019 (23.4% vs. 33.3%, Pearson’s chi-squared test, chi squared= 14.9, df = 1, *p* < 0.05) and 2020–2021 (18.6% vs. 24.6%, Pearson’s chi-squared test, chi squared= 4.16, df = 1, *p* < 0.05).

Moreover, a significantly reduced percentage of calls for patients admitted to the ED (ED group) was recorded during the pandemic compared to the pre-pandemic period (23.4% vs. 18.6%, Pearson’s chi-squared test, chi squared= 4.1, df= 1, *p* < 0.05). Similarly, a significantly reduced percentage of calls for patients not admitted to the ED (NED group) was recorded in 2020–2021 with respect to 2018–2019 (33.3% vs. 24.6%, Pearson’s chi-squared test, chi squared= 6.89, df = 1, *p* < 0.05).

For the 45–64 years of age group, a significant increase was observed between percentages of calls for both patients admitted to the ED (ED group) and percentages of calls for patients not admitted to the ED (NED group) both in 2018–2019 (23.1% vs. 33.5%, Pearson’s chi-squared test, chi squared = 16.39, df = 1, *p* = 0.05) and 2020–2021 (14.1% vs. 24.3%, Pearson’s chi-squared test, chi-squared = 17.17, df = 1, *p* < 0.05).

Moreover, a significant decrease in the percentage of calls for patients admitted to the ED (ED group) was recorded during the pandemic (23.1% vs. 14.1%, Pearson’s chi-squared test, chi squared= 17.8, df = 1, *p* < 0.05) compared with the 2-year pre-pandemic period (2018–2019). The same result was observed for the percentage of calls for patients not admitted to the ED (NED group) during the pandemic compared to the 2-year pre-pandemic period (33.5% vs. 24.3%, Pearson’s chi-squared test, chi squared = 9.14, df = 1, *p* < 0.05).

For the ≥65 years of age group, a significant reduction in the percentage of calls for patients admitted to the ED (ED group) in the COVID-19 period (2020–2021) compared to the 2-year pre-COVID-19 period (2018–2019) (28.2% vs. 15.4%, Pearson’s chi-squared test, chi squared= 5.24, df= 1, *p*< 0.05) was observed.

Because metadoxine (also referred to as pyridoxol L-2-pyrrolidone-5-carboxilate) is the primary pharmacological treatment for alcohol-related emergencies [9], we evaluated the percentage of calls for patients treated with metadoxine by ESs and admitted (ED group) or not admitted (NED group) to the ED per age group.

No significant change was observed when evaluating the percentage of calls for patients treated with metadoxine by ESs to be admitted (ED group) or not admitted (NED group) to the ED in the 2-year periods by age group, suggesting that the pandemic had no effect on either group (Table 6).

Moreover, irrespective of the group or the period under examination, a high percentage of calls for patients treated with metadoxine was observed in the 11–17 and 18–24 years of age groups, whereas slightly lower percentages were observed for all other age groups.

To fully evaluate the pharmacological treatment administered, we analyzed the number of other drugs used with or without metadoxine in both the ED and NED groups.

In the Methods section, we describe the single drugs belonging to each class as reported in Table 7A,B.

The data shown in Table 7 indicate the pattern of pharmacological treatment, other than metadoxine, of patients requiring ESs for alcohol-related issues admitted (ED group) or not admitted (NED group) to the ED.

Metadoxine was primarily administered in association with drugs acting on the gastrointestinal tract (pantoprazole, ranitidine, metoclopramide, acetylcysteine and levosulpiride), irrespective of age, period considered, and whether patients were or were not admitted to the ED (Table 7A,B).

Moreover, if metadoxine was not administered, drugs acting on the CNS (benzodiazepines, chlorpromazine, haloperidol, and clotiapine) were the most commonly used (Table 7A,B).

Interestingly, when patients required metadoxine, whether or not they were admitted to the ED, naloxone and/or flumazenil were administered, suggesting that polydrug abuse was suspected.

In the case of calls for the ED group that was pharmacologically treated, the administration of naloxone and/or flumazenil was recorded for calls of patients aged 25–44, 45–64, and ≥65 years, but not for calls regarding younger patients. This was less evident in calls for NED patients not treated with metadoxine.

Drugs acting on the respiratory system (salbutamol, corticosteroids, theophylline, oxygen, and chlorpheniramine) were administered primarily to ED patients admitted to the ED whether or not they were treated with metadoxine.

For “other” drugs (pain killers, antispasmodics, insulin, and glucagon), no clear pattern of use across periods, age groups, and whether patients were to be admitted to the ED or not admitted was observed.

## 4. Discussion

Our study showed that during the COVID-19 pandemic the percentage of alcohol-related calls to the emergency number “118” with respect to emergency calls for all reasons was significantly reduced (2018–2019: 1.68% vs. 2020–2021: 1.18%). Moreover, a reduction of approximately 30% in calls for alcohol-related reasons in 2020–2021 compared to 2018–2019 was observed (Table 1). This finding suggests a “forced” reduced consumption of alcoholic beverages, at least in some subjects, due to (a) economic problems (such as job loss); (b) reduced socialization, due to the legislative limitations, as Italians tend to be social drinkers; and (c) the perception of alcohol as a dangerous substance to be avoided with the contingent pandemic conditions [8,11]. However, a survey conducted between April and July 2020 investigating alcohol-consumption attitudes in individuals who already had problematic alcohol consumption showed an increase in both the quantity and frequency of drinking in most European countries, including Italy [12].

The reduced number of calls to ESs during the pandemic may have been due to a more significant reluctance to request first aid assistance services and transport to the ED for the fear of possible close contact with patients affected by COVID-19 [13]. This hypothesis is supported by our finding of an increase (35.04% to 41.17%) in emergency calls not ending in ED admission between the 2-year periods analyzed owing to patient refusal.

A more in-depth examination was performed to assess which age group was most affected by the reduction in emergency calls for alcohol-related issues. The most significant reduction was observed in the 25–44 years of age group (−41.4%). As alcohol abuse has been shown to be the primary risk factor for disability in subjects aged 25–49 years [14], it is alarming that the reduction in calls for alcohol-related issues was extremely high for this age group, which renounced ES assistance.

Interestingly, our findings seemingly demonstrate that some age groups are less likely to reduce the use of ESs for alcohol-related issues, because a low reduction in calls for the 11–17 years of age group and for subjects aged ≥65 years, which represent the most fragile subjects, was observed. Moreover, a low reduction in calls for subjects aged 45–64, who are more likely to be affected by other concomitant organic pathologies, largely of alcohol-related origin (Table 1), was observed [15,16,17].

Table 2 shows that the percentages of calls for patients with alcohol-related problems who were pharmacologically treated do not reach a third of the calls irrespective of whether patients were admitted to the ED or not admitted (Table 2), in agreement with the existing literature [9]. However, Table 2 and Table 5, detailing age groups, show that percentages of emergency calls for patients pharmacologically treated in the ED groups were always lower compared to percentages of calls for the NED groups, irrespective of the period analyzed. Moreover, during the pandemic, we observed a reduction in the percentage of calls for patients admitted to the ED compared to those in the pre-pandemic period. The same was observed for calls regarding NED patients. In particular, the lack of a reduction in calls for patients of 11–17 years of age who were admitted to the ED and pharmacologically treated during the pandemic agrees with the reported increase in risky alcohol consumption in adolescents and younger individuals (Table 5) [18].

These findings are parallel with the reduced calls coded green and yellow for alcohol-related issues (Table 3 and Table 4 detailing age groups).

Our findings are in agreement with the literature reporting reduced access to EDs in white and green codes [13]. Our study highlights that, during the pandemic, no inverse association was found for calls coded red in the ED group between pharmacological treatment and periods under examination (Table 3). These data are in agreement with a study that demonstrated an increase in alcohol consumption during the pandemic in participants with riskier alcohol behaviors [12].

With regard to the pharmacological treatment for acute alcohol intoxication, metadoxine is currently the primary effective drug in acute alcohol intoxication because it can accelerate ethanol clearance and improve symptoms [16,19]. In agreement with the literature, metadoxine was administered to nearly all patients aged 11–17 and 18–24 years with no difference between the ED and NED groups or in the period under examination (Table 6). These findings emphasize the importance of the accelerated elimination of alcohol in adolescents who are highly sensitive to the toxic effects of alcohol. However, metadoxine was administered to 52.4% and 77.9% of patients in all other age groups owing to its role in improving the symptoms of potentially life-threatening alcohol intoxication [9,16].

These results suggest that the following: (i) as pharmacological treatment is time-consuming, because of the preparation of the patient as the treatment is primarily administered intravenously, pharmacological treatment was reduced for less serious health conditions to reduce the duration of the intervention of the emergency team to ensure their prompt availability for further emergencies; (ii) in contrast, the increase in the percentage of subjects with medium severity codes treated pharmacologically and not admitted to the ED aimed to resolve the emergency on-site to avoid crowding hospital services; and (iii) the increase in the percentage of red codes of subjects treated pharmacologically and subsequently admitted to the ED aimed to reduce the time spent in the ED, in consideration of the long procedure required by the pharmacological treatment (primarily metadoxine).

Collectively, they indicate that the type of intervention, based on the severity of calls coded by the triage, documents the virtuosity of the service carried out by the 118 ES that carefully balanced the opposing requirements, which, however, required the commitment of the ES for alcohol-related emergencies and required strategies aimed at desaturating the ED.

Finally, as shown in Table 7A,B and in line with the existing literature, metadoxine was primarily administered in association with drugs acting on the gastrointestinal tract (proton pump inhibitors, H2-antagonists, metoclopramide, acetylcysteine and levosulpiride), but also with (i) drugs acting on the CNS (benzodiazepines and antipsychotics), (ii) those acting on the respiratory system (salbutamol, corticosteroids, theophylline, oxygen, and chlorpheniramine), and (iii) painkillers and antispasmodics, irrespective of the age group, the period considered, and whether patients were or were not admitted to the ED [16].

Moreover, when metadoxine was not administered, drugs acting on the CNS (benzodiazepines and antipsychotics) were the most commonly administered drugs. In this instance, we hypothesize that the ES had to treat a possible alcohol withdrawal syndrome, which likely occurred when prolonged heavy drinking was drastically reduced owing to the difficulty in maintaining drinking habits during the pandemic [16].

We recorded the administration of naloxone and/or flumazenil in combination with metadoxine to ED patients and less frequently to NED patients to treat polydrug abuse [9]. Interestingly, in cases without metadoxine, the administration of naloxone and/or flumazenil was recorded primarily in patients aged 25–44, 45–64, and ≥65 years, who were treated by the ED (Table 7A), suggesting that polydrug abusers ended in the ED.

We conclude that the ES is the first and only out-of-hospital service that a percentage of patients, 35.04% before the pandemic and 41.17% during the pandemic, will make use of in an alcohol-related emergency because they refuse to be admitted to the ED. Therefore, it is important to ensure the engagement of clinicians and nurses through telemedicine/telehealth to screen patients, particularly minors, with acute alcohol intoxication for underlying alcohol-use disorders, and to implement a multidisciplinary program to assist in reducing excessive drinking through combined psychosocial and pharmacological interventions [19,20,21].

Limitations: We recognize that the evaluation of the CIWA or agitation score is a useful tool to determine the severity of the alcohol intoxication or signs of withdrawal [22]. Unfortunately, no such parameter was recorded on site locations. Further studies should be conducted to evaluate the role of sex in acute alcohol intoxication during the COVID-19 pandemic and ES. This study primarily focused on the possible impact of the pandemic on emergency calls in specific age groups as well as ED and NED groups.

## 5. Conclusions

This study suggests the importance of developing a plan for public health with attention to risky alcohol consumption that requires the ES, which may be the first and only out-of-hospital service used by numerous individuals affected by alcohol-use disorders. This project should involve the engagement of ES clinicians and emergency doctors and nurses in screening patients, particularly minors, for acute alcohol intoxication and underlying alcohol-use disorders. Patients may be referred to a multidisciplinary program that combines psychosocial and pharmacological interventions to assist in reducing drinking.

## Figures and Tables

**Table 1:** Demographic characteristics and age distribution of alcohol-related calls between 2-year periods (2018–2019, pre-COVID-19 period; 2020–2021, COVID-19 period).

	Number of Calls		% Δ
Two-Year Period	2018–2019	2020–2021	
Total calls	3710	2601	−29.89
Age groups (years)			
11–17	179	141	−21.2
18–24	435	328	−24.6
25–44	1361	798	−41.4
45–64	1275	1008	−20.9
≥65	218	184	−15.6
not reported	242	142	−41.0
Gender			
female	780	629	−19.4
male	2908	1961	−32.6
not reported	22	11	

**Table 2:** Emergency calls for alcohol-related issues that ended in the emergency department (ED group) or not (NED group) for patients pharmacologically treated in 2-year periods (2018–2019, pre-COVID-19 period; 2020–2021, COVID-19 period).

	ED Group	NED Group
2018–2019	604/2408 (25.08%)	417/1301 (32.05%) *
2020–2021	261/1529 (17.07%) ^	269/1072 (25.09%) *,§

* *p* < 0.05, ED group vs. NED group in the same time period. ^ *p* < 0.05, ED group _2018–2019_ vs. ED group _2020–2021_. § *p* < 0.05, NED group _2018–2019_ vs. NED group _2020–2021_.

**Table 3:** Emergency calls coded “green” (non-critical), “yellow” (fairly critical), and “red” (very critical) for alcohol-related issues for patients pharmacologically treated who ended in the emergency department or not in 2-year periods (2018–2019, pre-COVID-19 period; 2020–2021, COVID-19 period).

Triage	ED Group		NED Group	
	2018–2019	2020–2021	2018–2019	2020–2021
green	289	84 ^	397	243 §
yellow	278	136 ^	20	25
red	37	41	0	1

^ *p* < 0.05, ED group _2018–2019_ vs. ED group _2020–2021_. § *p* < 0.05, NED group _2018–2019_ vs. NED group _2020–2021_.

**Table 4:** Emergency calls for alcohol-related issues, coded “green” (non-critical), “yellow” (fairly critical), and “red” (very critical) for patients pharmacologically treated who were admitted to the emergency department or not in the 2-year periods (2018–2019, pre-COVID-19 period; 2020–2021, COVID-19 period) by age groups.

Age Groups (Years)	Triage	ED Group		NED Group	
		2018–2019	2020–2021	2018–2019	2020–2021
11–17	green	17	1	25	13
	yellow	28	19	1	1
	red	2	9	0	0
18–24	green	47	14	46	42
	yellow	46	15	0	3
	red	3	8	0	0
25–44	green	108	30	145	77
	yellow	93	49	5	6
	red	12	7	0	0
45–64	green	84	26	149	90
	yellow	86	45	12	12
	red	14	12	0	0
≥65	green	14	4	27	17
	yellow	14	8	2	3
	red	5	4	0	1

**Table 5:** Emergency calls for alcohol-related issues for patients pharmacologically treated who were admitted to the emergency department or not in 2-year periods (2018–2019, pre-COVID-19 period; 2020–2021, COVID-19 period) by age groups.

Age Groups (Years)	Period	ED Group		NED Group	
11–17	2018–2019	47/123	(38.2%)	26/56	(46.4%)
	2020–2021	29/87	(33.3%)	14/54	(25.9%) §
18–24	2018–2019	96/283	(33.9%)	46/152	(30.3%)
	2020–2021	37/193	(19.2%) ^	45/135	(33.3%) *
25–44	2018–2019	213/909	(23.4%)	150/451	(33.3%) *
	2020–2021	86/461	(18.6%) ^	83/337	(24.6%) *,§
45–64	2018–2019	184/795	(23.1%)	161/480	(33.5%) *
	2020–2021	83/589	(14.1%) ^	102/419	(24.3%) *,§
≥65	2018–2019	33/117	(28.2%)	29/101	(28.7%)
	2020–2021	16/104	(15.4%) ^	21/80	(26.3%)

* *p* < 0.05 ED group vs. NED group in the same time period. ^ *p* < 0.05 ED group 2018–2019 vs. ED group 2020–2021. § *p* < 0.05 NED group 2018–2019 vs. NED group 2020–2021.

**Table 6:** Emergency calls for patients administered metadoxine by ES to be admitted or not admitted to the emergency department in 2-year periods (2018–2019, pre-COVID-19 period; 2020–2021, COVID-19 period) by age groups.

Age Groups (Years)	Period	ED Group	NED Group
11–17	2018–2019	43/47 (91.5%)	24/26 (92.3%)
	2020–2021	28/29 (96.6%)	13/14 (92.9%)
18–24	2018–2019	85/96 (88.5%)	39/46 (84.8%)
	2020–2021	32/37 (86.5%)	37/45 (82.2%)
25–44	2018–2019	166/213 (77.9%)	104/150 (69.3%)
	2020–2021	62/86 (72.1%)	64/83 (77.1%)
45–64	2018–2019	124/184 (67.4%)	107/161 (66.5%)
	2020–2021	60/83 (72.3%)	79/102 (77.5%)
>65	2018–2019	24/33 (72.7%)	22/29 (75.9%)
	2020–2021	10/16 (62.5%)	11/21 (52.4%)

**Table 7:** (**A**). Number of other medicines administered by ES to patients to be admitted to the emergency department in the 2-year periods (2018–2019, pre-COVID-19 period; 2020–2021, COVID-19 period) by age groups. (**B**). Number of other medicines administered by ESs to patients not admitted to the emergency department in the 2-year periods (2018–2019, pre-COVID-19 period; 2020–2021, COVID-19 period) by age groups.

**(A)**											
		**In Association with Metadoxine**					**Without Metadoxine**				
**Age Groups** **(Years)**	**Period**	**CNS**	**Gastro**	**Resp**	**Nal/Flu**	**Others**	**CNS**	**Gastro**	**Resp**	**Nal/Flu**	**Others**
11–17	2018–2019	3	21	0	1	1	0	3	0	0	0
	2020–2021	0	8	2	0	0	0	0	1	0	0
18–24	2018–2019	6	48	7	7	1	4	3	5	0	0
	2020–2021	1	8	3	4	2	2	3	0	0	2
25–44	2018–2019	26	41	4	12	10	32	16	8	4	11
	2020–2021	9	19	7	3	4	17	7	2	0	7
45–64	2018–2019	18	27	3	9	9	27	15	18	7	14
	2020–2021	14	8	2	6	5	11	8	5	2	11
≥65	2018–2019	1	8	3	0	3	1	2	4	1	4
	2020–2021	1	0	1	1	1	4	1	2	1	1
**(B)**											
		**In Association with Metadoxine**					**Without Metadoxine**				
**Age Groups (Years)**	**Period**	**CNS**	**Gastro**	**Resp**	**Nal/Flu**	**Others**	**CNS**	**Gastro**	**Resp**	**Nal/Flu**	**Others**
11–17	2018–2019	1	18	0	0	0	0	1	1	0	1
	2020–2021	0	8	0	0	0	0	2	0	0	0
18–24	2018–2019	8	25	0	1	3	3	5	0	0	0
	2020–2021	5	16	2	2	1	7	2	0	2	0
25–44	2018–2019	10	66	2	7	10	38	15	1	0	6
	2020–2021	15	20	0	2	6	16	3	0	1	4
45–64	2018–2019	18	49	1	5	13	30	19	6	0	14
	2020–2021	11	27	2	7	10	12	10	7	0	10
≥65	2018–2019	1	14	0	0	0	3	6	0	0	6
	2020–2021	1	3	0	0	2	4	7	3	0	7

CNS (Central Nervous System): benzodiazepines, chlorpromazine, haloperidol and clotiapine. GI (gastrointestinal): pantoprazole, ranitidine, metoclopramide, acetylcysteine and levosulpiride. RS (Respiratory System): salbutamol, corticosteroids, theophylline, oxygen and chlorpheniramine. Nal/Flu: naloxone and/or flumazenil. Other: pain killers, antispasmodics, insulin and glucagon.

## Data Availability

Data are unavailable due to privacy or ethical restrictions.
